# Role of the Inflammasome Pathway According to the Expression of Proteins and Genetic Polymorphisms in COVID-19 Patients

**DOI:** 10.3390/ijms26209993

**Published:** 2025-10-14

**Authors:** Thiago Rodrigues dos Santos, Lucas Baena Carstens, Leonardo Vinícius Barbosa, Mariana Collete, Natan de Araujo, Caroline Busatta Vaz de Paula, Marina Luise Viola Azevedo, Ana Clara de Almeida, Seigo Nagashima, Lucia de Noronha, Cleber Machado-Souza

**Affiliations:** 1 Postgraduate Program of Biotechnology Applied to Child and Adolescent, Faculdades Pequeno Príncipe, Instituto de Pesquisa Pelé Pequeno Príncipe, Av. Silva Jardim, 1632, Curitiba 80250-200, PR, Brazil; thiagorsantos.ippp@gmail.com (T.R.d.S.);; 2 Postgraduate Program of Health Sciences, School of Medicine and Life Sciences, Pontifical Catholic University of Paraná—PUCPR, Rua Imaculada Conceição, 1155-Prado Velho, Curitiba 80215-901, PR, Brazil; lbc017@gmail.com (L.B.C.); natandearaujo@outlook.com.br (N.d.A.); lucia.noronha@pucpr.br (L.d.N.); 3 Laboratory of Experimental Pathology, School of Medicine and Life Sciences, Pontifical Catholic University of Paraná—PUCPR, Rua Imaculada Conceição, 1155-Prado Velho, Curitiba 80215-901, PR, Brazil; busatta.caroline@pucpr.br (C.B.V.d.P.); marina.azevedo@pucpr.br (M.L.V.A.); ana.calmeida@pucpr.br (A.C.d.A.); seigo.nagashima@pucpr.br (S.N.)

**Keywords:** SARS-CoV-2, COVID-19 waves, inflammasome, piroptose, polymorphisms

## Abstract

COVID-19 severity is frequently linked to exacerbated inflammation, with the inflammasome pathway playing a key role in activating inflammatory interleukins. This observational *post-mortem* study evaluated the expression of inflammasome-associated molecules in patients who died from COVID-19 during the second wave. Minimally invasive autopsies were performed on patients from the first (n = 24) and second (n = 18) waves. Lung tissue samples underwent immunohistochemical staining for ACE-2, TLR-4, NF-κB, TNF-α, NOX4, NLRP3, ASC, CASPASE-1, IL-1β, IL-18, GSDMD, and CASPASE-9. Additionally, genetic polymorphisms within inflammasome-related genes were assessed via real-time polymerase chain reaction. Lung tissue expressions of TLR-4, NLRP3, and IL-18 were significantly higher in patients from the second wave compared to those from the first, with expression levels of 26.3 versus 12.1, 13.9 versus 6.4, and 25.6 versus 3.8, respectively. The A allele at rs4648090 of *NFKB1* and the T allele at rs317155 of *NOX4* were associated with increased corresponding protein expression by factors of 5.1 and 8.9, respectively. Notably, IL-18 demonstrated substantial immunological relevance, correlating strongly with elevated expression linked to these genetic variants in second wave cases. These findings suggest that the inflammasome pathway harbors biologically meaningful molecules implicated in severe COVID-19, meriting further investigation for their potential as diagnostic or therapeutic targets.

## 1. Introduction

Several host-related risk factors have been identified as contributors to severe disease and increased mortality in coronavirus infections [1,2]. Current research indicates that the most severe COVID-19 cases typically involve clinical features such as lymphopenia, hypoalbuminemia, elevated lactate dehydrogenase, increased C-reactive protein, ferritin, and D-dimer levels. These alterations often accompany an exaggerated inflammatory response termed the “cytokine storm” [3,4,5]. Elevated cytokine concentrations contribute to the pathogenesis of severe acute respiratory syndrome (SARS) caused by SARS-CoV-2, where the virus triggers intense inflammation that can exacerbate clinical decline and increase the risk of fatality.

Central to inflammation is the activation of the inflammasome, a multi-protein intracellular complex that orchestrates inflammatory responses and induces a form of programmed cell death known as pyroptosis [6,7]. The inflammasome responds to diverse stimuli, including pathogen-associated molecular patterns (PAMPs), damage-associated molecular patterns (DAMPs), nucleic acids, and pore-forming toxins. Key molecules such as NLRP3, ASC, pro-caspase-1, pro-IL-1β, and IL-18 have established roles in inflammasome activation [8,9,10]. Extensive research has implicated the inflammasome pathway in numerous diseases, and during the COVID-19 pandemic, new and repurposed drugs targeting this pathway, including disulfiram and biologics like Anakinra^®^ and Canakinumab^®^, were evaluated for modulating the inflammatory cascade related to pyroptosis and endothelial dysfunction [11,12,13]. These molecular components represent promising candidates for further study.

Genetic variation across the human genome, particularly single-nucleotide polymorphisms (SNPs), can significantly influence gene expression and function. While many SNPs exert minimal effects, select polymorphisms may affect disease susceptibility, clinical outcomes, and response to treatment [14,15,16]. Consequently, genetic variants may serve as important components in future biomarker discovery and understanding individual risk profiles.

Biomarkers—both genetic and protein—offer measurable insights into biological states, facilitating diagnosis, prognosis, and prediction of treatment responses. The expression of inflammasome-related proteins may be modulated by specific polymorphisms, underscoring the potential of these molecules as indicators of severity and therapeutic targets in COVID-19. Given that the second wave of COVID-19 in Brazil yielded higher mortality, this study hypothesizes that differential expression and polymorphisms in inflammasome pathway genes among deceased patients could highlight molecules of biological relevance for future pandemics. Accordingly, this study aims to investigate the relationship between SNPs in inflammasome-associated genes and protein expression levels in *post-mortem* lung tissues from COVID-19 patients, using the second wave as a reference for outcome analysis.

## 2. Results

### 2.1. Sociodemographic and Clinical Information

Table 1 compares demographic and clinical variables between the first and second COVID-19 waves. Patients in the first wave were significantly older, with a median age of 72.5 years versus 55.5 years in the second wave (*p* < 0.0001). Mechanical ventilation duration was longer in the second wave group (median 14.5 days) compared to the first wave (9.5 days, *p* = 0.040). No significant difference was observed in hospital stay length until death (*p* = 0.430).

### 2.2. Immunohistochemistry Findings

**Figure 1:**
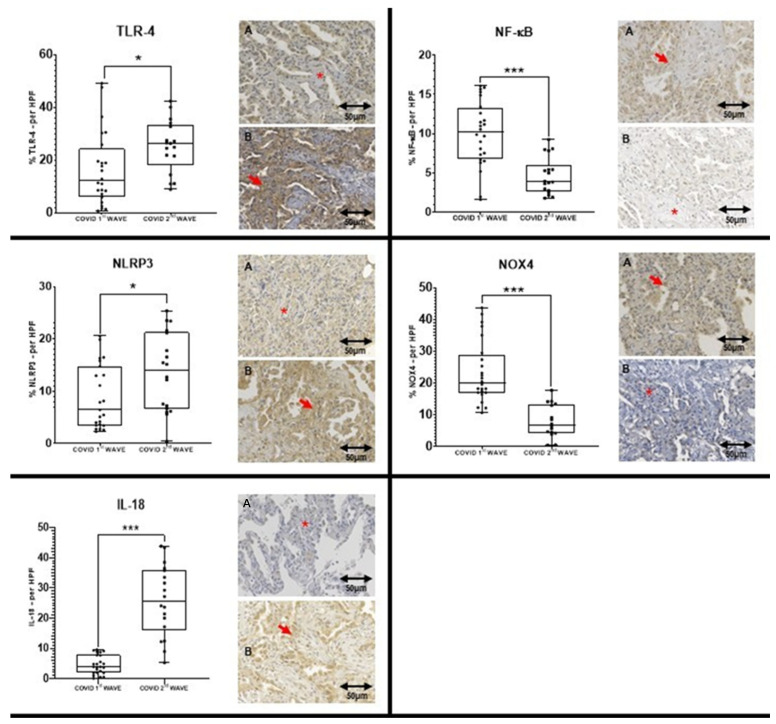
Comparison between the COVID 1st WAVE (**A**) and COVID 2nd WAVE (**B**) regarding TLR-4, NLRP3, IL-18, NF-kB and NOX4 expressions. The left Panel shows the expression of three markers that are significantly higher in the COVID 2nd WAVE group compared to the COVID 1st WAVE group. The right Panel shows the expression of two markers that are significantly higher in the COVID 1st WAVE group compared to the COVID 2nd WAVE group. The symbol “***” in the graph means *p* ≤ 0.001, while “*” in the graph means *p* ≤ 0.05 and in the image appoint to an area without immunoexpression. The red arrows are appointed to an area with immunoexpression. The slide images were obtained at 40× magnification.

Immunohistochemistry revealed significantly elevated expressions of TLR-4, NLRP3, and IL-18 in lung tissue from second wave patients compared to the first (median percentages: 26.3 vs. 12.1, 13.9 vs. 6.4, and 25.6 vs. 3.8, respectively). After Bonferroni correction for multiple comparisons, only IL-18 maintained statistical significance. Other markers such as ACE-2 and GSDMD showed higher expression in the second wave but without statistical significance, while NF-κB and NOX4 expression were lower in the second wave (Table 2, Figure 1).

### 2.3. Genotyping Findings

Table 3 summarizes genotype frequencies across COVID-19 waves. Significant differences were noted with elevated homozygosity for *ACE2* rs4646188 AA (*p* = 0.000), rs4646156 AA (*p* = 0.028), and rs2048683 GG (*p* = 0.028) polymorphisms in the second wave. For *TLR4* rs10759932 CT genotype, a higher frequency was observed in the second wave (*p* = 0.000). After Bonferroni correction (*p* < 0.001), significance persisted for *ACE2* rs4646188 and *TLR4* rs10759932. No significant results were found in the first and second wave of patients in grouping (dominant and recessive analysis (see Supplementary Tables S1 and S2).

Further genotype clustering analyses linked the *NFKB1* rs4648090 AA genotype and *NOX4* rs317155 TT + TC cluster with higher protein expression in second wave lung tissues (*p* = 0.022 and *p* = 0.008, respectively), although these associations lost significance after strict correction. IL-18-related polymorphisms (rs1946518, rs187238) showed expression trends with biological plausibility despite lacking statistical significance (Table 4).

The main analysis of this study was conducted to verify a possible association between genotypes and high tissue expression using genotype clustering models (dominant and recessive) in the first and second waves. The AA genotype for rs4648090 [G/A] of *NFKB1* (*p* = 0.022) and the TT + TC cluster for rs317155 [T/C] of the *NOX4* gene (*p* = 0.008) were significantly associated with the highest expression of the proteins encoded by these genes in the cohort of patients affected by the second wave of COVID-19 (Table 4); however, before the Bonferroni correction (*p* < 0.001), this association was lost. Considering that in the first round of analysis only IL-18 remained significant after statistical correction, the SNPs in the *IL18* gene, despite not presenting statistical significance, could acquire some biological plausibility. The highest expressions of IL-18 can be associated with the GG + GT (rs1946518 G/T), and CC + CG (rs187238 C/G) grouping genotypes, respectively. No significant results were found in the first wave of patients in this analysis (see Supplementary Tables S3 and S4).

Multivariate analyses were performed, but no significance was found for any of the variables.

## 3. Discussion

This study aimed to further elucidate marked differences in the expression of inflammasome-related proteins and associated genetic polymorphisms between COVID-19 fatal cases from the first and second pandemic waves in Brazil. Our findings contribute to understanding the molecular underpinnings that may explain the increased severity and mortality characterizing the second wave.

The COVID 1st WAVE group exhibited a higher mean age with mostly older patients. In contrast, the COVID 2nd WAVE group predominantly comprised younger patients, which aligns with the findings reported in the extant literature [17]. The age is a major risk factor for severe COVID-19, since older patients are more vulnerable due to immune senescence and chronic low-grade inflammation [18], every ten years the risk of death rises by 1.5 times [2,19]. Despite the higher risk, the second wave of COVID-19 in Brazil was three times deadlier than the first, affecting the entire population [20]. This demonstrates that other major aspects were involved in the severity of the disease. The clinical variables not addressed in relation to the 1st and 2nd waves in this sample were not part of the analyses of this article as they have already been the subject of discussion in other articles of the group [21,22,23,24,25,26,27,28,29,30].

Immunohistochemical analysis (Table 2) revealed significantly elevated lung tissue expression of TLR-4, NLRP3, and IL-18 during the second wave, with IL-18 expression showing robust statistical significance even after stringent Bonferroni correction. This pronounced IL-18 elevation aligns with the literature recognizing IL-18 as a critical pro-inflammatory cytokine driving pulmonary inflammation and systemic cytokine storm in severe COVID-19 [31,32]. The enhanced TLR-4 expression mirrors its role as a pattern recognition receptor that can be activated by SARS-CoV-2 components and oxidized phospholipids, stimulating innate immune responses and perpetuating inflammation [33,34,35,36]. Similarly, increased NLRP3 levels reflect inflammasome assembly and activation, consistent with its established contribution to IL-1β and IL-18 maturation and subsequent pyroptotic cell death in infected tissues [8,9,10,37,38].

ACE-2 and GSDMD also demonstrated higher expression in the second wave, although without statistical significance. ACE-2 plays a dual role as the viral entry receptor and regulator of inflammatory pathways [39,40,41,42,43,44,45,46]. Elevated ACE-2 expression in *post-mortem* tissues may indicate enhanced viral tropism or compensatory mechanisms during later disease stages. GSDMD mediates pyroptotic membrane pore formation, further amplifying inflammasome downstream effects [47,48]. Conversely, NF-κB and NOX4 levels were paradoxically reduced in the second wave. Given NF-κB’s role in transcriptional regulation of inflammatory genes and NOX4’s involvement in reactive oxygen species generation, their reduced expression may represent negative feedback or cell exhaustion phenomena arising during sustained inflammation [8,9,49,50,51,52,53,54].

Genetic analysis (Table 3) uncovered higher frequencies of homozygous genotypes for *ACE2* rs4646188 AA, rs4646156 AA, and rs2048683 GG in second wave patients, polymorphisms previously implicated in susceptibility to severe pulmonary and cardiovascular pathologies [42,43,44,45,46]. The enrichment of *TLR4* rs10759932 CT genotype aligns with reports linking this variant to altered immune responses in chronic lung diseases [35,55]. Clustering analyses (Table 4) further demonstrated that *NFKB1* rs4648090 AA and *NOX4* rs317155 TT + TC genotypes associate with increased protein expression, underscoring the genetic influence on inflammasome pathway activity. Though statistical significance was lost after correction, these results present biologically plausible mechanisms by which host genetics exacerbate inflammasome-mediated injury.

The observed shift toward younger patients with prolonged ventilation during the second wave points to changes in clinical phenotype potentially driven by viral evolution and immune landscape remodeling [56,57]. Enhanced inflammasome activation likely exacerbates lung tissue damage through sustained cytokine release and pyroptosis, contributing to increased mortality despite demographic differences [58,59].

Our data help support a model in which inflammasome activation may contribute as a central factor in the severe pathology of COVID-19, intensified in the second wave by both increased cytokine expression and host genetic predisposition. This reinforces the promise of inflammasome components, particularly IL-18, as future biomarkers and potential therapeutic targets [11,12,13,60]. Future interventions aimed at modulating inflammasome activation have the potential to improve outcomes, especially for genetically susceptible individuals.

This study’s limitations include sample size constraints with associated power limitations, especially for genetic analyses, and the inability to temporally track longitudinal changes due to the *post-mortem* study design. Nonetheless, the integrated immunohistochemical and genetic approach offers valuable insights into host–pathogen interactions driving COVID-19 mortality across epidemic waves.

## 4. Materials and Methods

### 4.1. Study Population

This study was conducted following the Declaration of Helsinki and approved by the National Research Ethics Committee (CONEP; Project ID: 30188020.7.1001.0020). Families of deceased COVID-19 patients provided informed consent for minimally invasive *post-mortem* lung biopsies. The cohort consisted of 42 patients admitted to the intensive care unit at Hospital Marcelino Champagnat, Curitiba, Brazil. Samples included 24 patients from the first COVID-19 wave (June–August 2020) and 18 from the second wave (April–May 2021). All patients had SARS-CoV-2 confirmed by RT-PCR performed on nasopharyngeal swabs during hospitalization. Patients with negative SARS-CoV-2 tests or without consent were excluded.

### 4.2. Tissue Processing and Immunohistochemistry

*Post-mortem* lung biopsy fragments were formalin-fixed, paraffin-embedded, and assembled into tissue microarray (TMA) blocks. Sections (4 μm) were mounted on positively charged slides. Immunohistochemistry was performed using primary monoclonal antibodies against ACE-2, TLR-4, NF-κB, TNF-α, NOX4, NLRP3, ASC, CASPASE-1, IL-1β, IL-18, GSDMD, and CASPASE-9. Primary antibody incubation occurred overnight at 2–8 °C. Visualization employed horseradish peroxidase conjugated polymer detection with diaminobenzidine substrate, followed by Harris hematoxylin counterstain. Positive control reactivity confirmed assay specificity. The authors will indicate only those markers that show significantly higher tissue expression in the second wave group, as the first wave was recently reported for these samples [26].

### 4.3. Morphometric Analysis

Slides were digitally scanned (Axio Scan Z1, Carl Zeiss—ZEISS, Jena, Germany) producing approximately 500 high-power field images per sample. Non-representative images (e.g., bronchioles, artifacts) were excluded. This process was performed by a single researcher, ensuring the homogeneity of the selection. Thirty representative images per case were randomly selected for quantification. Image ProPlus^®^ software version 4 (Media Cybernetics, Rockville, MD, USA) semi-automatically measured immunostained area, expressed as a percentage of total tissue area, averaged per patient.

### 4.4. DNA Extraction and Genotyping

Genomic DNA was extracted from paraffin-embedded tissue using phenol-chloroform deparaffinization. DNA purity was assessed spectrophotometrically (OD 260/280). SNP selection targeted (Available at: https://snpinfo.niehs.nih.gov accessed on 30 July 2024) twelve inflammasome pathway genes (*ACE2*, *TLR4*, *NFKB1*, *TNFA*, *NOX4*, *NLRP3*, *ASC*, *CASP1*, *IL1B*, *IL18*, *GSDMD*, *CASP9*) based on SNPinfo database (parameters tuned for a CEU population proxy for the southern Brazilian population. The Brazilian population is heterogeneous and has overlapping genotypes due to miscegenation, but the authors used the CEU population due to its greater proximity to the population of southern Brazil where the study was carried out. Tag SNPs with functional relevance from literature were included. Genotyping was performed by real-time PCR using TaqMan^®^ probes on an Applied Biosystems 7500 system (Foster City, CA, USA). Genotypes were called automatically based on fluorescent signal ratios; homozygous genotypes produce single fluorophore signals, and heterozygotes display dual signals. Ten percent of samples were re-run to assess reproducibility.

### 4.5. Statistical Analysis

Categorical variables were represented as counts and percentages, continuous variables as medians with ranges. Differences between waves were assessed by Pearson’s chi-square, Fisher’s exact, or Mann–Whitney tests as appropriate. Logistic regression examined genotype-phenotype associations. Bonferroni adjustment corrected for multiple comparisons. Statistical significance was set at *p* < 0.05 before correction. For the multivariate analysis, the binary logistic regression model was used and included clinical variables, IHQ and SNP for each gene. A simplified Bonferroni correction was applied, based on dividing the significant *p*-value by the number of variables analyzed in the expression and genotypes. Analyses leveraged SPSS v20.0 (IBM, Chicago, IL, USA) and GraphPad Prism 8.0 (La Jolla, CA, USA).

## 5. Conclusions

With the COVID-19 pandemic now concluded, our study offers valuable insights into the molecular mechanisms underlying fatal outcomes during its course, particularly in the second epidemic wave. We demonstrated that lung tissues from patients who died in this wave exhibit increased expression of inflammasome-associated proteins, especially IL-18, TLR-4, and NLRP3, and that specific genetic polymorphisms in *NFKB1* and *NOX4* correlate with enhanced inflammasome activation. These results emphasize the role of host genetics and immune dysregulation in determining COVID-19 severity and mortality.

Our findings provide a theoretical foundation for developing targeted biomarkers and therapeutic strategies aimed at inflammasome components, which may have broader applications in managing hyperinflammation in future infectious or inflammatory diseases. The molecular distinctions identified between epidemic waves underline the dynamic interplay between viral evolution and host response, underscoring the importance of ongoing monitoring and personalized interventions beyond the pandemic context.

In summary, enhanced inflammasome activation driven by molecular and genetic factors highlights important aspects of fatal COVID-19 in the second wave, highlighting IL-18 and related genetic markers (rs4648090 *NFKB1* and rs317155 *NOX4*) as molecules of key interest for prognostic and therapeutic consideration in future severe respiratory diseases.

## Figures and Tables

**Table 1:** Comparison between COVID-19 (1st WAVE) and COVID-19 (2nd WAVE) according to demographic and clinical findings.

Characteristics	COVID 1st WAVE (n = 24)	COVID 2nd WAVE (n = 18)	*p*-Value
Age ^a^	72.5 (46.0–93.0)	55.5 (30.0–83.0)	0.000 **
Female ^b^	9 (37.5)	3 (16.6)	0.180 *
Male ^b^	15 (62.5)	15 (83.3)	
Mechanical Ventilation ^c^	9.5 (0.0–36.0)	14.5 (5.0–28.0)	0.040 ***
Time from admission to death ^c^	13.0 (1.0–39.0)	14.5 (5.0–28.0)	0.430 ***

^a^ time in years. ^c^ time in days. ^b^ absolute number and (frequency). * Fisher’s exact test. ** Unpaired *t*-test. *** Mann–Whitney test.

**Table 2:** Comparison between COVID-19 (1st WAVE) and COVID-19 (2nd WAVE) groups according to immunohistochemical findings.

Markers	COVID 1st WAVE (n = 24)	COVID 2nd WAVE (n = 18)	*p*-Value *
ACE-2	1.3 (0.1–24.2)	1.8 (0.2–6.5)	0.865
TLR-4	12.1 (0.6–49.1)	26.3 (8.9–42.3)	0.011
NF-κB	10.2 (1.6–16.1)	3.9 (1.8–9.3)	0.000
TNF-α	11.4 (2.2–26.0)	7.2 (1.2–13.5)	0.010
NOX4	20.1 (10.7–43.7)	6.8 (0.5–17.8)	0.000
NLRP3	6.4 (2.3–20.6)	13.9 (0.4–25.4)	0.025
ASC	14.7 (0.1–29.7)	4.0 (0.6–21.3)	0.002
Caspase-1	20.6 (12.2–32.9)	7.5 (0.1–40.9)	0.001
IL-1β	18.6 (6.1–39.8)	13.5 (1.7–30.7)	0.409
IL-18	3.8 (0.1–9.5)	25.6 (5.2–43.8)	0.000
GSDMD	11.7 (5.1–61.6)	17.1 (5.0–24.5)	0.365
Caspase-9	15.1 (1.3–34.1)	0.5 (0.1–2.4)	0.000

Median (minimum–maximum). * Mann–Whitney test. *p*-value valid after Bonferroni correction (*p* < 0.004).

**Table 3:** Distribution of genotype frequencies in COVID-19 groups (1st and 2nd waves).

Gene—Tag SNPs dbSNP ID * [Allele Variation] **	COVID Groups	Homozygous 1/1 ^†^	Heterozygous 1/2 ^†^	Homozygous 2/2 ^†^	*p*-Value ^‡^
*ACE2*		AA	AG	GG	
rs4646188 [A/G]	1st wave	0 (0.0)	20 (83.3)	4 (16.7)	0.000
	2nd wave	16 (88.9)	1 (5.6)	1 (5.6)	
*ACE2*		CC	CG	GG	
rs879922 [C/G]	1st wave	5 (20.8)	2 (8.3)	17 (70.8)	0.436
	2nd wave	7 (38.9)	1 (5.6)	10 (55.6)	
*ACE2*		AA	AT	TT	
rs4646156 [A/T]	1st wave	3 (12.5)	4 (16.7)	17 (70.8)	0.028
	2nd wave	9 (50.0)	2 (11.1)	7 (38.9)	
*ACE2*		TT	TG	GG	
rs2048683 [T/G]	1st wave	17 (70.8)	4 (16.7)	3 (12.5)	0.028
	2nd wave	7 (38.9)	2 (11.1)	9 (50.0)	
*TLR4*		AA	AG	GG	
rs4986790 [A/G]	1st wave	17 (85.0)	3 (15.0)	0 (0.0)	0.232
	2nd wave	18 (100.0)	0 (0.0)	0 (0.0)	
*TLR4*		CC	CT	TT	
rs4986791 [C/T]	1st wave	17 (85.0)	3 (15.0)	0 (0.0)	0.232
	2nd wave	18 (100.0)	0 (0.0)	0 (0.0)	
*TLR4*		CC	CT	TT	
rs10759932 [C/T]	1st wave	12 (66.7)	6 (33.3)	0 (0.0)	0.000
	2nd wave	0 (0.0)	18 (100.0)	0 (0.0)	
*TLR4*		CC	CG	GG	
rs11536889 [C/G]	1st wave	1 (4.2)	12 (50.0)	11 (45.8)	0.196
	2nd wave	0 (0.0)	5 (27.8)	13 (72.2)	
*NFKB1*		AA	AT	TT	
rs3821958 [A/T]	1st wave	9 (37.5)	14 (58.3)	1 (4.2)	0.509
	2nd wave	5 (27.8)	13 (72.2)	0 (0.0)	
*NFKB1*		GG	GA	AA	
rs4648090 [G/A]	1st wave	0 (0.0)	7 (30.4)	16 (69.6)	0.340
	2nd wave	1 (5.6)	3 (16.7)	14 (77.8)	
*NFKB1*		CC	CT	TT	
rs4648022 [C/T]	1st wave	21 (87.5)	3 (12.5)	0 (0.0)	1.000
	2nd wave	15 (83.3)	3 (16.7)	0 (0.0)	
*TNFA*		GG	GA	AA	
rs3093661 [G/A]	1st wave	21 (87.5)	3 (12.5)	0 (0.0)	1.000
	2nd wave	16 (88.9)	2 (11.1)	0 (0.0)	
*TNFA*		AA	AG	GG	
rs3093662 [A/G]	1st wave	18 (75.0)	6 (25.0)	0 (0.0)	0.481
	2nd wave	12 (66.7)	5 (27.8)	1 (5.6)	
*NOX4*		GG	GA	AA	
rs9299894 [G/A]	1st wave	20 (87.0)	3 (13.0)	0 (0.0)	1.000
	2nd wave	16 (88.9)	2 (11.1)	0 (0.0)	
*NOX4*		TT	TA	AA	
rs7939071 [T/A]	1st wave	3 (12.5)	10 (41.7)	11 (45.8)	0.819
	2nd wave	2 (11.1)	6 (33.3)	10 (55.6)	
*NOX4*		TT	TC	CC	
rs317155 [T/C]	1st wave	8 (34.8)	13 (56.5)	2 (8.7)	0.184
	2nd wave	3 (16.7)	10 (55.6)	5 (27.8)	
*NOX4*		CC	CG	GG	
rs7925520 [C/G]	1st wave	10 (41.7)	10 (41.7)	4 (16.7)	0.841
	2nd wave	6 (33.3)	9 (50.0)	3 (16.7)	
*NLRP3*		TT	TC	CC	
rs4612666 [T/C]	1st wave	15 (65.2)	7 (30.4)	1 (4.3)	0.537
	2nd wave	9 (50.0)	7 (38.9)	2 (11.1)	
*NLRP3*		GG	GC	CC	
rs10754558 [G/C]	1st wave	11 (45.8)	11 (45.8)	2 (8.3)	0.596
	2nd wave	6 (33.3)	9 (50.0)	3 (16.7)	
*NLRP3*		AA	AG	GG	
rs2027432 [A/G]	1st wave	2 (8.7)	8 (34.8)	13 (56.5)	0.419
	2nd wave	0 (0.0)	6 (33.3)	12 (66.7)	
*ASC*		CC	CT	TT	
rs8056505 [C/T]	1st wave	15 (62.5)	6 (25.0)	3 (12.5)	0.705
	2nd wave	13 (72.2)	4 (22.2)	1 (5.6)	
*CASP1*		TT	TC	CC	
rs530537 [T/C]	1st wave	4 (16.7)	9 (37.5)	11 (45.8)	0.792
	2nd wave	2 (11.1)	6 (33.3)	10 (55.6)	
*CASP1*		GG	GA	AA	
rs572687 [G/A]	1st wave	0 (0.0)	5 (20.8)	19 (79.2)	0.060
	2nd wave	0 (0.0)	0 (0.0)	18 (100.0)	
*CASP1*		GG	GA	AA	
rs571593 [G/A]	1st wave	0 (0.0)	5 (20.8)	19 (79.2)	0.060
	2nd wave	0 (0.0)	0 (0.0)	18 (100.0)	
*CASP1*		AA	AG	GG	
rs2282659 [A/G]	1st wave	15 (62.5)	6 (25.0)	3 (12.5)	0.679
	2nd wave	11 (61.1)	6 (33.3)	1 (5.6)	
*CASP1*		CC	CT	TT	
rs501192 [C/T]	1st wave	19 (79.2)	5 (20.8)	0 (0.0)	0.060
	2nd wave	18 (100.0)	0 (0.0)	0 (0.0)	
*IL1B*		CC	CT	TT	
rs1143633 [C/T]	1st wave	11 (45.8)	8 (33.3)	5 (20.8)	0.225
	2nd wave	7 (38.9)	10 (55.6)	1 (5.6)	
*IL1B*		AA	AG	GG	
rs3136558 [A/G]	1st wave	17 (70.8)	5 (20.8)	2 (8.3)	0.343
	2nd wave	12 (66.7)	6 (33.3)	0 (0.0)	
*IL1B*		GG	GA	AA	
rs1143634 [G/A]	1st wave	18 (75.0)	4 (16.7)	2 (8.3)	0.088
	2nd wave	10 (55.6)	8 (44.4)	0 (0.0)	
*IL18*		GG	GT	TT	
rs1946518 [G/T]	1st wave	8 (33.3)	15 (62.5)	1 (4.2)	0.180
	2nd wave	6 (33.3)	8 (44.4)	4 (22.2)	
*IL18*		CC	CG	GG	
rs187238 [C/G]	1st wave	15 (65.2)	8 (34.8)	0 (0.0)	0.126
	2nd wave	10 (55.6)	5 (27.8)	3 (16.7)	
*GDSMD*		CC	CT	TT	
rs1545536 [C/T]	1st wave	18 (75.0)	6 (25.0)	0 (0.0)	0.481
	2nd wave	12 (66.7)	5 (27.8)	1 (5.6)	
*GDSMD*		AA	AG	GG	
rs2305492 [A/G]	1st wave	13 (54.2)	11 (45.8)	0 (0.0)	0.052
	2nd wave	8 (44.4)	6 (33.3)	4 (22.2)	
*CASP9*		CC	CT	TT	
rs4646012 [C/T]	1st wave	7 (36.8)	9 (47.4)	3 (15.8)	0.057
	2nd wave	6 (40.0)	2 (13.3)	7 (46.7)	
*CASP9*		GG	GA	AA	
rs4646063 [G/A]	1st wave	0 (0.0)	8 (33.3)	16 (66.7)	0.501
	2nd wave	1 (5.6)	6 (33.3)	11 (61.1)	

* SNP identification based on NCBI dbSNP. ** Allele variation: 1 wild allele; 2 polymorphic allele; ^†^ Absolute number and frequency (percentage). For each genotype, the percentage values were described in the line; ^‡^ Logistic regression for *p* value. The sample number is different within variables due to technical failures in the tests performed. *p*-value valid after Bonferroni correction (*p* < 0.001).

**Table 4:** Immunohistochemical expression in genotyping groups (dominant model) in COVID 2nd wave.

Gene—Tag SNPs dbSNP ID * [Allele Variation] ^†^	Dominant Model	Tissue Expression	*p*-Value ^‡^
*ACE2*	AA + AG	1.6 (0.2–6.5)	0.838
rs4646188 A/G	GG	1.9 (1.9–1.9)	
*ACE2*	CC + CG	1.8 (0.4–6.5)	0.853
rs879922 C/G	GG	1.5 (0.2–6.1)	
*ACE2*	AA + AT	1.9 (0.2–6.1)	0.807
rs4646156 A/T	TT	1.6 (0.4–6.5)	
*ACE2*	TT + TG	1.6 (0.4–6.5)	0.788
rs2048683 T/G	GG	1.9 (0.2–6.1)	
*TLR4*	CC + CT	26.3 (8.9–42.3)	N/A
rs10759932 C/T	TT	0.0 (0.0–0.0)	
*TLR4*	CC + CG	24.9 (11.0–34.0)	0.346
rs11536889 C/G	GG	26.4 (8.9–42.3)	
*NFKB1*	AA + AT	3.9 (1.8–9.2)	N/A
rs3821958 A/T	TT	0.0 (0.0–0.0)	
*NFKB1*	GG + GA	2.1 (1.8–3.6)	0.022
rs4648090 G/A	AA	5.1 (2.5–9.2)	
*TNFA*	AA + AG	7.1 (1.2–13.5)	0.625
rs3093662 A/G	GG	8.4 (8.4–8.4)	
*NOX4*	TT + TA	6.8 (0.5–14.3)	0.810
rs7939071 T/A	AA	7.1 (0.6–17.8)	
*NOX4*	TT + TC	8.9 (4.0–17.8)	0.008
rs317155 T/C	CC	2.5 (0.5–4.6)	
*NOX4*	CC + CG	7.2 (0.5–17.8)	0.613
rs7925520 C/G	GG	5.7 (0.6–13.5)	
*NLRP3*	TT + TC	14.1 (5.6–25.4)	0.260
rs4612666 T/C	CC	7.8 (0.4–15.1)	
*NLRP3*	GG + CC	12.7 (0.4–25.4)	0.742
rs10754558 G/C	CC	16.5 (6.7–21.6)	
*NLRP3*	AA + AG	13.9 (6.0–23.4)	0.920
rs2027432 A/G	GG	14.4 (0.4–25.4)	
*ASC*	CC + CT	4.1 (0.6–21.3)	0.585
rs8056505 C/T	TT	3.8 (3.8–3.8)	
*CASP1*	TT + TC	5.7 (0.1–32.2)	0.565
rs530537 T/C	CC	8.0 (0.1–40.9)	
*CASP1*	AA + AG	8.2 (0.1–40.9)	0.355
rs2282659 A/G	GG	0.1 (0.1–0.1)	
*IL1B*	CC + CT	13.2 (1.7–30.7)	0.167
rs1143633 C/T	TT	29.9 (29.9–29.9)	
*IL1B*	AA + AG	13.5 (1.7–30.7)	N/A
rs3136558 A/G	GG	0.0 (0.0–0.0)	
*IL1B*	GG + GA	13.5 (1.7–30.7)	N/A
rs1143634 G/A	AA	0.0 (0.0–0.0)	
*IL18*	GG + GT	28.2 (12.2–43.8)	0.157
rs1946518 G/T	TT	16.3 (5.2–35.8)	
*IL18*	CC + CG	27.2 (9.0–43.8)	0.506
rs187238 C/G	GG	23.6 (5.2–35.8)	
*GDSMD*	CC + CT	17.2 (5.0–24.5)	0.524
rs1545536 C/T	TT	14.3 (14.3–14.3)	
*GDSMD*	AA + AG	17.1 (5.0–24.5)	0.834
rs2305492 A/G	GG	18.0 (12.4–23.9)	
*CASP9*	CC + CT	0.6 (0.2–2.4)	0.801
rs4646012 C/T	TT	0.7 (0.1–2.3)	
*CASP9*	GG + GA	0.5 (0.1–0.9)	0.198
rs4646063 G/A	AA	0.7 (0.1–2.4)	

* SNP identification based on NCBI dbSNP; ^†^ Allele variation [wild allele/polymorphic allele]; ^‡^ Mann–Whitney U Test for *p* value. N/A = Not Available. Polymorphisms that were not associated with expression were removed from the table. The sample number is different within variables due to technical failures in the tests performed. *p*-value valid after Bonferroni correction (*p* < 0.001).

## Data Availability

The original contributions presented in the study are included in the article/Supplementary Materials. Further inquiries can be directed to the corresponding authors.

## Supplementary Materials

The following supporting information can be downloaded at: https://www.mdpi.com/article/10.3390/ijms26209993/s1.

## Abbreviations

The following abbreviations are used in this manuscript:

ACE2	Angiotensin-converting enzyme 2
ASC	Apoptosis-associated speck-like protein containing a CARD
CARD	Caspase Recruitment Domain
CONEP	National Research Ethics Committee
DAB	3,3-diaminobenzidine
DAD	Diffuse alveolar damage
DNA	Deoxyribonucleic acid
GSDMD	Gasdermin D
HRP	Horseradish peroxidase
ICU	Intensive Care Unit
IHQ	Immunohistochemistry
IL	Interleucin
NETs	Neutrophil extracellular traps
NF-κB	Nuclear factor kappa B
NK	Natural killer
NLRP3	Nod (Nucleotide-binding oligomerization domain)-, LRR (Leucine-rich repeat)- and pyrin domain-containing protein 3
PAMP	Pathogen-Associated Molecular Patterns
RNA	Ribonucleic acid
ROS	Reactive oxygen species
RT-PCR	Real-Time Polymerase Chain Reaction
SARS-CoV-2	Severe Acute Respiratory Syndrome Coronavirus 2
SNP	Single-Nucleotide Polymorphism
TLR	Toll-Like receptor
TMA	Tissue Microarray
TNF-α	Tumor Necrosis Factor-alpha
