# Supplementary material for: Role of the Inflammasome Pathway According to the Expression of Proteins and Genetic Polymorphisms in COVID-19 Patients
Source: Int J Mol Sci. 2025 Oct 14;26(20):9993. doi: 10.3390/ijms26209993 (PMC12564305; doi:10.3390/ijms26209993)
Supplement: Supplementary file 1 [file ijms-26-09993-s001.zip › ijms-3881585-supplementary.pdf]

Suppl table S1. Genotyping dominant model in COVID-19 1st and 2nd waves.

Gene - Tag SNPs dbSNP ID * Allele variation†	Dominant model	COVID 1st WAVE (n=18)	COVID 2nd WAVE (n=18)	*p*-Value‡
ACE2	AA+AG	20 (83.3)	17 (94.4)	0.710
rs4646188 [A/G]	GG	4 (16.7)	1 (5.6)	
ACE2	CC+CG	7 (29.2)	8 (44.4)	0.307
rs879922 [C/G]	GG	17 (70.8)	10 (55.6)	
ACE2	AA+AT	7 (29.2)	11 (61.1)	0.038
rs4646156 [A/T]	TT	17 (70.8)	7 (38.9)	
ACE2	TT+TG	21 (87.5)	9 (50.0)	0.014
rs2048683 [T/G]	GG	3 (12.5)	9 (50.0)	
TLR4	CC+CT	18 (100.0)	18 (100.0)	-
rs10759932 [C/T]	TT	-	-	
TLR4	CC+CG	13 (54.2)	5 (27.8)	0.087
rs11536889 [C/G]	GG	11 (45.8)	13 (72.2)	
NFKB1	AA+AT	23 (95.8)	18 (100.0)	1.000
rs3821958 [A/T]	TT	1 (4.2)	0 (0.0)	
NFKB1	GG+GA	7 (30.4)	4 (22.2)	0.726
rs4648090 [G/A]	AA	16 (69.6)	14 (77.8)	
TNFA	AA+AG	24 (100.0)	17 (94.4)	0.429
rs3093662 [A/G]	GG	0 (0.0)	1 (5.6)	
NOX4	TT+TA	13 (54.2)	8 (44.4)	0.533
rs7939071 [T/A]	AA	11 (45.8)	10 (55.6)	
NOX4	TT+TC	21 (91.3)	13 (72.2)	0.209
rs317155 [T/C]	CC	2 (8.7)	5 (27.8)	
NOX4	CC+CG	20 (83.3)	15 (83.3)	1.000
rs7925520 [C/G]	GG	4 (16.7)	3 (16.7)	
NLRP3	TT+TC	22 (95.7)	16 (88.9)	0.573
rs4612666 [T/C]	CC	1 (4.3)	2 (11.1)	
NLRP3	GG+CC	22 (91.7)	15 (83.3)	0.636
rs10754558 [G/C]	CC	2 (8.3)	3 (16.7)	
NLRP3	AA+AG	10 (43.5)	6 (33.3)	0.509
rs2027432 [A/G]	GG	13 (56.5)	12 (66.7)	
ASC	CC+CT	21 (87.5)	17 (94.4)	0.623
rs8056505 [C/T]	TT	3 (12.5)	1 (5.6)	
CASP1	TT+TC	13 (54.2)	8 (44.4)	0.756
rs530537 [T/C]	CC	11 (45.8)	10 (55.6)	
CASP1	AA+AG	21 (87.5)	17 (94.4)	0.623
rs2282659 [A/G]	GG	3 (12.5)	1 (5.6)	
IL1B	CC+CT	19 (79.2)	17 (94.4)	0.214
rs1143633 [C/T]	TT	5 (20.8)	1 (5.6)	
IL1B	AA+AG	22 (91.7)	18 (100.0)	0.498
rs3136558 [A/G]	GG	2 (8.3)	0 (0.0)	
IL1B	GG+GA	22 (91.7)	18 (100.0)	0.498
rs1143634 [G/A]	AA	2 (8.3)	0 (0.0)	
IL18	GG+GT	23 (95.8)	14 (77.8)	0.146
rs1946518 [G/T]	TT	1 (4.2)	4 (22.2)	
IL18	CC+CG	23 (100.0)	15 (83.3)	0.077
rs187238 [C/G]	GG	0 (0.0)	3 (16.7)	
GDSMD	CC+CT	24 (100.0)	17 (94.4)	0.429
rs1545536 [C/T]	TT	0 (0.0)	1 (5.6)	
GDSMD	AA+AG	24 (100.0)	14 (77.8)	0.027
rs2305492 [A/G]	GG	0 (0.0)	4 (22.2)	
CASP9	CC+CT	16 (84.2)	8 (53.3)	0.068
rs4646012 [C/T]	TT	3 (15.8)	7 (46.7)	
CASP9	GG+GA	8 (33.3)	7 (38.9)	0.710

rs4646063 [G/A]	AA	16 (66.7)	11 (61.1)

Legend: *SNP identification based on NCBI dbSNP. †Absolute number and frequency (percentage). For each genotype, the percentage values were described in the rows; ‡ Pearson Chi test for *p* value. Polymorphisms that were not associated with expression were removed from the table. The sample number is different within variables due to technical failures in the tests performed.

Suppl table S2. Genotyping recessive model in COVID-19 1st and 2nd waves.

Tag SNPs dbSNP ID * Allele variation†	Recessive model	COVID 1st WAVE	COVID 2nd WAVE	*p*-Value‡
ACE2	AG+GG	24 (100.0)	2 (11.1)	0.000
rs4646188 [A/G]	AA	0 (0.0)	16 (88.9)	
ACE2	CG+GG	19 (79.2)	11 (61.1)	0.200
rs879922 [C/G]	CC	5 (20.8)	7 (38.9)	
ACE2	AT+TT	21 (87.5)	9 (50.0)	0.014
rs4646156 [A/T]	AA	3 (12.5)	9 (50.0)	
ACE2	TG+GG	7 (29.2)	11 (61.1)	0.038
rs2048683 [T/G]	TT	17 (70.8)	7 (38.9)	
TLR4	CT+TT	6 (33.3)	18 (100.0)	0.000
rs10759932 [C/T]	CC	12 (66.7)	0 (0.0)	
TLR4	CG+GG	23 (95.8)	18 (100.0)	1.000
rs11536889 [C/G]	CC	1 (4.2)	0 (0.0)	
NFKB1	AT+TT	15 (62.5)	13 (72.2)	0.508
rs3821958 [A/T]	AA	9 (37.5)	5 (27.8)	
NFKB1	GA+AA	23 (100.0)	17 (94.4)	0.439
rs4648090 [G/A]	GG	0 (0.0)	1 (5.6)	
TNFA	AG+GG	6 (25.0)	6 (33.3)	0.554
rs3093662 [A/G]	AA	18 (75.0)	12 (66.7)	
NOX4	TA+AA	21 (87.5)	16 (88.9)	1.000
rs7939071 [T/A]	TT	3 (12.5)	2 (11.1)	
NOX4	TC+CC	15 (65.2)	15 (83.3)	0.291
rs317155 [T/C]	TT	8 (34.8)	3 (16.7)	
NOX4	CG+GG	14 (58.3)	12 (66.7)	0.582
rs7925520 [C/G]	CC	10 (41.7)	6 (33.3)	
NLRP3	TC+CC	8 (34.8)	9 (50.0)	0.326
rs4612666 [T/C]	TT	15 (65.2)	9 (50.0)	
NLRP3	GC+CC	13 (54.2)	12 (66.7)	0.414
rs10754558 [G/C]	GG	11 (45.8)	6 (33.3)	
NLRP3	AG+GG	21 (91.3)	18 (100.0)	0.495
rs2027432 [A/G]	AA	2 (8.7)	0 (0.0)	
ASC	CT+TT	8 (34.7)	5 (27.8)	0.607
rs8056505 [C/T]	AA	15 (65.3)	13 (72.2)	
CASP1	TC+CC	20 (83.3)	16 (88.9)	0.685
rs530537 [T/C]	TT	4 (16.7)	2 (11.1)	
CASP1	AG+GG	9 (37.5)	7 (38.9)	0.927
rs2282659 [A/G]	AA	15 (62.5)	11 (61.1)	
IL1B	CT+TT	13 (54.2)	11 (61.1)	0.653
rs1143633 [C/T]	CC	11 (45.8)	7 (38.9)	
IL1B	AG+GG	7 (29.2)	6 (33.3)	0.773
rs3136558 [A/G]	AA	17 (70.8)	12 (66.7)	
IL1B	GA+AA	6 (25.0)	8 (44.4)	0.186
rs1143634 [G/A]	GG	18 (75.0)	10 (55.6)	
IL18	CG+GG	8 (34.8)	8 (44.4)	0.529
rs187238 [C/G]	CC	15 (65.2)	10 (55.6)	
GDSMD	CT+TT	6 (25.0)	6 (33.3)	0.554
rs1545536 [C/T]	CC	18 (75.0)	12 (66.7)	
GDSMD	AG+GG	11 (45.8)	10 (55.6)	0.533

rs2305492 [A/G]	AA	13 (54.2)	8 (44.4)	
CASP9	CT+TT	12 (63.2)	9 (60.0)	0.851
rs4646012 [C/T]	CC	7 (36.8)	6 (40.0)	
CASP9	GA+AA	24 (100.0)	17 (94.4)	0.429
rs4646063 [G/A]	GG	0 (0.0)	1 (5.6)	

Legend: *SNP identification based on NCBI dbSNP. †Absolute number and frequency (percentage). For each genotype, the percentage values were described in the rows; ‡ Pearson Chi test for *p* value. Polymorphisms that were not associated with expression were removed from the table. The sample number is different within variables due to technical failures in the tests performed.

Suppl table S3. Immunohistochemical expression in genotyping groups (dominant and recessive models) in COVID-19 1st wave.

Gene - Tag SNPs dbSNP ID * Allele variation†	Dominant model	Tissue expression	*p*-Value‡	Recessive model	Tissue expression	*p*-Value‡
ACE2	AA+AG	2.3 (0.2 – 24.2)	0.337	AG+GG	1.3 (0.2 – 24.2)	N/A
rs4646188 [A/G]	GG	0.8 (0.2 – 1.0)		AA	0.0 (0.0 – 0.0)	
ACE2	CC+CG	1.2 (0.3 – 5.2)	0.225	CG+GG	2,3 (0.2 – 24,2)	0.372
rs879922 [C/G]	GG	3.2 (0.2 – 24.2)		CC	1,2 (0.3 – 5.2)	
ACE2	AA+AT	0.7 (0.3 – 5.2)	0.202	AT+TT	1.4 (0.2 – 24.2)	0.570
rs4646156 [A/T]	TT	3.2 (0.2 – 24.2)		AA	0.5 (0.3 – 5.2)	
ACE2	TT+TG	1.4 (0.2 – 24.2)	0.570	TG+GG	0.7 (0.3 – 5.2)	0.202
rs2048683 [T/G]	GG	0.5 (0.3 – 5.2)		TT	3.2 (0.2 – 24.2)	
TLR4	AA+AG	0.0 (0.0 – 0.0)	N/A	AG+GG	0.0 (0.0 – 0.0)	N/A
rs4986790 [A/G]	GG	0.0 (0.0 – 0.0)		AA	0.0 (0.0 – 0.0)	
TLR4	CC+CT	0 (0.0 – 0.0)	N/A	CT+TT	0 (0.0 – 0.0)	N/A
rs4986791 [C/T]	TT	0 (0.0 – 0.0)		CC	0 (0.0 – 0.0)	
TLR4	CC+CT	11.2 (0,7 – 49.1)	N/A	CT+TT	14.1 (5.9 – 49.1)	0.381
rs10759932 [C/T]	TT	0.0 (0.0 – 0.0)		CC	11.2 (0.7 – 47.6)	
TLR4	CC+CG	12.8 (1.4 – 49.1)	0.604	CG+GG	11.3 (0.7 – 49.1)	0.974
rs11536889 [C/G]	GG	8.4 (0.7 – 47.6)		CC	16.0 (16.0 – 16.0)	
NFKB1	AA+AT	10.6 (1.6 – 16.1)	0.377	AT+TT	9.9 (5.2 – 16.1)	0.475
rs3821958 [A/T]	TT	6.3 (6.3 – 6.3)		AA	10.6 (1.6 – 15.9)	
NFKB1	GG+GA	9.9 (6.3 – 15.3)	0.922	GA+AA	10.6 (1.9 – 16.1)	N/A
rs4648090 [G/A]	AA	11.1 (1.9 – 16.1)		GG	0.0 (0.0 – 0.0)	
NFKB1	CC+CT	0.0 (0.0 – 0.0)	N/A	CT+TT	0.0 (0.0 – 0.0)	N/A
rs4648022 [C/T]	TT	0.0 (0.0 – 0.0)		CC	0.0 (0.0 – 0.0)	
TNFA	GG+GA	0.0 (0.0 – 0.0)	N/A	GA+AA	0.0 (0.0 – 0.0)	N/A
rs3093661 [G/A]	AA	0.0 (0.0 – 0.0)		GG	0.0 (0.0 – 0.0)	
TNFA	AA+AG	11.4 (2.2 – 26.0)	N/A	AG+GG	11.4 (6.2 – 19.7)	0.675
rs3093662 [A/G]	GG	0.0 (0.0 – 0.0)		AA	11.4 (2.2 – 26.0)	
NOX4	GG+GA	0.0 (0.0 – 0.0)	N/A	GA+AA	0.0 (0.0 – 0.0)	N/A
rs9299894 [G/A]	AA	0.0 (0.0 – 0.0)		GG	0.0 (0.0 – 0.0)	
NOX4	TT+TA	20.5 (14.0 – 43.7)	0.465	TA+AA	19.6 (10.7 – 41.7)	0.485
rs7939071 [T/A]	AA	18.3 (10.7 – 37.9)		TT	20.5 (17.1 – 43.7)	
NOX4	TT+TC	18.3 (10.7 – 43.7)	0.257	TC+CC	19.6 (12.2 – 43.7)	0.748
rs317155 [T/C]	CC	31.1 (20.5 – 41.7)		TT	19.6 (10.7 – 37.9)	
NOX4	CC+CG	19.0 (10.7 – 43.7)	0.508	CG+GG	19.9 (10.7 – 41.7)	0.533
rs7925520 [C/G]	GG	26.4 (17.6 – 35.1)		CC	20.1 (12.3 – 43.7)	
NLRP3	TT+TC	6.5 (2.6 – 20.6)	0.872	TC+CC	7.2 (3.7 – 14.8)	0.456
rs4612666 [T/C]	CC	8.2 (8.2 – 8.2)		TT	8.6 (2.6 – 20.6)	
NLRP3	GG+CC	6.5 (2.3 – 20.6)	0.867	GC+CC	12.1 (2.3 – 20.6)	0.065
rs10754558 [G/C]	CC	9.6 (2.8 – 16.3)		GG	5.1 (2.6 – 15.8)	
NLRP3	AA+AG	6.0 (2.8 – 16.3)	0.358	AG+GG	6.7 (2.6 – 20.6)	0.664
rs2027432 [A/G]	GG	9.5 (2.6 – 20.6)		AA	7.3 (6.5 – 8.2)	
ASC	CC+CT	15.8 (0.1 – 29.7)	0.211	CT+TT	8.7 (5.1 – 16.3)	0.073
rs8056505 [C/T]	TT	8.3 (7.9 – 13.0)		AA	19.2 (0.1 – 29.7)	
CASP1	TT+TC	23.3 (12.2 – 32.9)	0.877	TC+CC	20.6 (12.2 – 32.9)	0.977
rs530537 [T/C]	CC	20.0 (14.9 – 32.5)		TT	22.8 (15.6 – 29.7)	
CASP1	GG+GA	0.0 (0.0 – 0.0)	N/A	GA+AA	0.0 (0.0 – 0.0)	N/A
rs572687 [G/A]	AA	0.0 (0.0 – 0.0)		GG	0.0 (0.0 – 0.0)	
CASP1	GG+GA	0 (0.0 – 0.0)	N/A	GA+AA	0.0 (0.0 – 0.0)	N/A
rs571593 [G/A]	AA	0 (0.0 – 0.0)		GG	0.0 (0.0 – 0.0)	
CASP1	AA+AG	20.1 (12.2 – 32.9)	0.561	AG+GG	20.0 (12.2 – 32.9)	0.443
rs2282659 [A/G]	GG	29.0 (15.6 – 29.7)		AA	23.3 (14.9 – 32.5)	
CASP1	CC+CT	0.0 (0.0 – 0.0)	N/A	CT+TT	0.0 (0.0 – 0.0)	N/A
rs501192 [C/T]	TT	0.0 (0.0 – 0.0)		CC	0.0 (0.0 – 0.0)	
IL1B	CC+CT	18.2 (6.1 – 39.9)	0.318	CT+TT	19.3 (7.9 – 31.9)	0.586

rs1143633 [C/T]	TT	19.3 (7.9 – 19.5)		CC	18.2 (6.1 – 39.9)	
IL1B	AA+AG	18.6 (6.1 – 39.9)	0.930	AG+GG	16.1 (6.1 – 31.9)	0.764
rs3136558 [A/G]	GG	19.0 (15.9 – 22.1)		AA	19.1 (8.2 – 39.9)	
IL1B	GG+GA	18.6 (6.1 – 39.9)	0.930	GA+AA	16.0 (8.2 – 31.9)	0.807
rs1143634 [G/A]	AA	19.0 (15.9 – 22.1)		GG	19.2 (6.1 – 39.9)	
IL18	GG+GT	3.9 (0,13 – 9.5)	0.767	GT+TT	4.8 (0.13 – 9.5)	0.144
rs1946518 [G/T]	TT	3.6 (3.6 – 3.6)		GG	2.7 (0.56 – 9.3)	
IL18	CC+CG	3.9 (0.13 – 9.5)	N/A	CG+GG	4.8 (1.8 – 9.5)	0.311
rs187238 [C/G]	GG	0 (0.0 – 0.0)		CC	3.7 (0.13 – 9.3)	
GDSMD	CC+CT	11.7 (5.1 – 61.6)	N/A	CT+TT	31.2 (8.0 – 58.0)	0.114
rs1545536 [C/T]	TT	0.0 (0.0 – 0.0)		CC	11.1 (5.1 – 61.6)	
GDSMD	AA+AG	11.7 (5.1 – 61.6)	N/A	AG+GG	12.4 (5.1 – 61.6)	0.524
rs2305492 [A/G]	GG	0 (0.0 – 0.0)		AA	11.7 (7.2 – 39.2)	
CASP9	CC+CT	15.8 (1.3 – 34.1)	0.746	CT+TT	14.3 (1.3 – 28.4)	0.147
rs4646012 [C/T]	TT	18.5 (4.0 – 23.1)		CC	17.9 (9.7 – 34.1)	
CASP9	GG+GA	14.5 (4.0 – 23.1)	0.377	GA+AA	15.1 (1.3 – 34.1)	N/A
rs4646063 [G/A]	AA	16.7 (1.3 – 34.1)		GG	0 (0.0 – 0.0)	

Legend: *SNP identification based on NCBI dbSNP; [a] Median (minimum-maximum) morphometry; ‡ Mann–Whitney test. N/A = Not Available.

Suppl table S4. Immunohistochemical expression in genotyping groups (recessive model) in COVID 2nd wave.

Gene - Tag SNPs dbSNP ID* Allele variation†	Recessive model	Tissue expression	*p*-Value‡
ACE2	AG+GG	3.1 (1.9 – 4.2)	0.600
rs4646188 [A/G]	AA	1.6 (0.2 – 6.5)	
ACE2	CG+GG	1.9 (0.2 – 6.1)	0.807
rs879922 [C/G]	CC	1.6 (0.4 – 6.5)	
ACE2	AT+TT	1.6 (0.4 – 6.5)	0.788
rs4646156 [A/T]	AA	1.9 (0.2 – 6.1)	
ACE2	TG+GG	1.9 (0.2 – 6.1)	0.807
rs2048683 [T/G]	TT	1.6 (0.4 – 6.5)	
TLR4	CT+TT	26.3 (8.9 – 42.3)	N/A
rs10759932 [C/T]	CC	0.0 (0.0 – 0.0)	
TLR4	CG+GG	26.3 (8.9 – 42.3)	N/A
rs11536889 [C/G]	CC	0.0 (0.0 – 0.0)	
NFKB1	AT+TT	4.0 (1.8 – 8.1)	0.725
rs3821958 [A/T]	AA	3.8 (2.8 – 9.3)	
NFKB1	GA+AA	4.0 (1.8 – 9.2)	0.667
rs4648090 [G/A]	GG	3.6 (3.6 – 3.6)	
TNFA	AG+GG	7.3 (1.2 – 8.6)	0.256
rs3093662 [A/G]	AA	7.5 (4.0 – 13.5)	
NOX4	TA+AA	7.2 (0.5 – 17.8)	0.761
rs7939071 [T/A]	TT	6.4 (6.4 – 6.4)	
NOX4	TC+CC	6.8 (0.5 – 17.8)	0.593
rs317155 [T/C]	TT	10.0 (5.7 – 14.2)	

NOX4	CG+GG	5.7 (0.6 – 14.3)	0.233
rs7925520 [C/G]	CC	13.0 (0.5 – 17.8)	
NLRP3	TC+CC	15.1 (0.4 – 25.4)	0.934
rs4612666 [T/C]	TT	12.2 (6.0 – 23.5)	
NLRP3	GC+CC	12.4 (0.4 – 25.4)	0.288
rs10754558 [G/C]	GG	18.1 (7.2 – 23.5)	
NLRP3	AG+GG	13.9 (0.4 – 25.4)	N/A
rs2027432 [A/G]	AA	0.0 (0.0 – 0.0)	
ASC	CT+TT	3.9 (0.6 – 18.0)	0.664
rs8056505 [C/T]	AA	4.1 (1.3 – 21.3)	
CASP1	TC+CC	8.7 (0.1 – 40.9)	0.277
rs530537 [T/C]	TT	1.4 (0.1 – 2.7)	
CASP1	AG+GG	8.2 (0.1 – 32.2)	0.801
rs2282659 [A/G]	AA	6.8 (0.1 – 40.9)	
IL1B	CT+TT	13.2 (1.7 – 30.7)	0.751
rs1143633 [C/T]	CC	20.3 (2.2 – 26.4)	
IL1B	AG+GG	16.7 (1.7 – 30.7)	0.968
rs3136558 [A/G]	AA	13.5 (2.0 – 29.9)	
IL1B	GA+AA	13.2 (1.7 – 30.7)	0.585
rs1143634 [G/A]	GG	19.6 (2.0 – 29.9)	
IL18	GT+TT	25.6 (5.2 – 43.8)	0.846
rs1946518 [G/T]	GG	25.3 (12.2 – 38.5)	
IL18	CG+GG	21.8 (5.2 – 35.8)	0.210
rs187238 [C/G]	CC	30.4 (9.0 – 43.8)	
GDSMD	CT+TT	16.4 (5.0 – 20.5)	0.137
rs1545536 [C/T]	CC	18.7 (11.7 – 24.5)	
GDSMD	AG+GG	20.5 (11.7 – 24.5)	0.082
rs2305492 [A/G]	AA	16.4 (5.0 – 20.1)	
CASP9	CT+TT	0.7 (0.1 – 2.3)	0.952
rs4646012 [C/T]	CC	0.6 (0.2 – 2.4)	
CASP9	GA+AA	0.6 (0.1 – 2.4)	0.542
rs4646063 [G/A]	GG	0.3 (0.3 – 0.3)	

* SNP identification based on NCBI dbSNP; a Median (minimum-maximum) morphometry; ‡ Mann–Whitney test. N/A = Not Available. Polymorphisms that were not associated with expression were removed from the table. The sample number is different within variables due to technical failures in the tests performed. *p*-value valid after Bonferroni correction ($p<0.001$).
